# Supplementary material for: FAM83H Expression Is Associated with Tumor-Infiltrating PD1-Positive Lymphocytes and Predicts the Survival of Breast Carcinoma Patients
Source: Diagnostics (Basel). 2023 Sep 15;13(18):2959. doi: 10.3390/diagnostics13182959 (PMC10529262; doi:10.3390/diagnostics13182959)
Supplement: Supplementary file 1 [file diagnostics-13-02959-s001.zip › diagnostics-2576027-supplementary.pdf]

Supplementary materials

FAM83H expression is associated with tumor-infiltrating PD1-positive lymphocytes and predicts the survival of breast carcinoma patients

Ji Eun Choi [1,*,†], Ae Ri Ahn [2,†], Junyue Zhang [2], Kyoung Min Kim [2,3,4], Ho Sung Park [2,3,4], Ho Lee [5], Myoung Ja Chung [2,3,4], Woo Sung Moon [2,3,4] and Kyu Yun Jang [2,3,4,*]

[1] Department of Pathology, Chungnam National University Sejong Hospital, Sejong 30099, Republic of Korea
[2] Department of Pathology, Jeonbuk National University Medical School, Jeonju 54896, Republic of Korea; xoxoyool@naver.com (A.R.A.); yuezai123@naver.com (J.Z.); kmkim@jbnu.ac.kr (K.M.K.); hspark@jbnu.ac.kr (H.S.P.); mjchung@jbnu.ac.kr (M.J.C.); mws@jbnu.ac.kr (W.S.M.)
[3] Research Institute of Clinical Medicine, Jeonbuk National University, Jeonju 54896, Republic of Korea
[4] Research Institute, Jeonbuk National University Hospital, Jeonju 54896, Republic of Korea
[5] Department of Forensic Medicine, Jeonbuk National University Medical School, Jeonju 54896, Republic of Korea; foremed@jbnu.ac.kr
* Correspondence: b612elf@gmail.com (J.E.C.); kyjang@jbnu.ac.kr (K.Y.J.)
† These authors contributed equally to this work.

Supplementary Table S1. Clinical variables and the expression of FAM83H and PD1 in BCAs.

**Characteristics**		**No.**	**FAM83H-Nu**		**FAM83H-Cy**		**PD1**	
			Positive	*p*	Positive	*p*	Positive	*p*
Age, y	< 50	136	60 (44%)	0.042	71 (52%)	0.067	52 (38%)	0.120
	≥50	62	37 (60%)		41 (66%)		31 (50%)	
Tumor stage	I	40	17 (43%)	0.008	24 (60%)	0.148	14 (35%)	0.416
	II	122	54 (44%)		63 (52%)		51 (42%)	
	III and IV	36	26 (72%)		25 (69%)		18 (50%)	
T category of stage	1	62	28 (45%)	0.236	37 (60%)	0.423	20 (32%)	0.076
	2	120	58 (48%)		64 (53%)		58 (48%)	
	3 and 4	16	11 (69%)		11 (69%)		5 (31%)	
Lymph node metastasis	Absence	107	43 (40%)	0.007	57 (53%)	0.310	41 (38%)	0.265
	Presence	91	54 (59%)		55 (60%)		42 (46%)	
Distant metastatic relapse	Absence	156	63 (40%)	< 0.001	82 (53%)	0.029	55 (35%)	< 0.001
	Presence	42	34 (81%)		30 (71%)		28 (67%)	
Histologic type	NST	190	95 (50%)	0.166	110 (58%)	0.066	79 (42%)	0.636
	Lobular	8	2 (25%)		2 (25%)		4 (50%)	
Histologic grade	1	55	19 (35%)	0.007	20 (36%)	< 0.001	23 (42%)	0.012
	2	96	47 (49%)		58 (60%)		32 (33%)	
	3	47	31 (66%)		34 (72%)		28 (60%)	
Tubule and gland formation	1	31	10 (32%)	0.029	10 (32%)	0.007	14 (45%)	0.157
	2	70	31 (44%)		46 (66%)		23 (33%)	
	3	97	56 (58%)		56 (58%)		46 (47%)	
Nuclear pleomorphism	1	11	4 (36%)	0.002	3 (27%)	< 0.001	5 (45%)	0.330
	2	69	23 (33%)		25 (36%)		24 (35%)	
	3	118	70 (59%)		84 (71%)		54 (46%)	
Mitoses/10 HPF	0-9	127	58 (46%)	0.151	67 (53%)	0.060	48 (38%)	0.241
	10-19	33	15 (45%)		17 (52%)		15 (45%)	
	> 19	38	24 (63%)		28 (74%)		20 (53%)	
HER2	Negative	133	53 (40%)	< 0.001	65 (49%)	0.002	53 (40%)	0.399
	Positive	65	44 (68%)		47 (72%)		30 (46%)	
ER	Negative	66	41 (62%)	0.009	45 (68%)	0.020	33 (50%)	0.103
	Positive	132	56 (42%)		67 (51%)		50 (38%)	
PR	Negative	89	51 (57%)	0.034	59 (66%)	0.013	41 (46%)	0.285
	Positive	109	46 (42%)		53 (49%)		42 (39%)	
PD1	Negative	115	43 (37%)	< 0.001	56 (49%)	0.009		
	Positive	83	54 (65%)		56 (67%)			
FAM83H-Cy	Negative	86	13 (15%)	< 0.001			26.8 ± 5.5*	0.099**
	Positive	112	84 (75%)				40.4 ± 5.8*	
FAM83H-Nu	Negative	101					24.6 ± 4.8*	0.012**
	Positive	97					44.8 ± 6.5*	

*The number of PD1-positive cells, mean ± standard error. **Student’s t-test. Abbreviations: FAM83H-Nu, nuclear FAM83H; FAM83H-Cy, cytoplasmic FAM83H; NST, no special type; ER, estrogen receptor; PR, progesterone receptor.
